# Supplementary material for: Factors associated with weighing a child at birth: Evidence from 16 sub-Saharan African countries
Source: PLoS One. 2025 Jul 22;20(7):e0328463. doi: 10.1371/journal.pone.0328463 (PMC12282927; doi:10.1371/journal.pone.0328463)
Supplement: S2 Appendix — (DOCX) [file pone.0328463.s002.docx]

S2 Appendix: Collinearity diagnostics for the independent variable

| Variable | VIF | TI |
| --- | --- | --- |
| Child sex | 1.00 | 0.99 |
| Birth type | 1.01 | 0.98 |
| Age | 2.33 | 0.43 |
| Education level | 1.55 | 0.64 |
| Marital Status | 1.13 | 0.88 |
| Residence | 1.43 | 0.70 |
| Parity | 2.53 | 0.40 |
| Health decisions | 1.08 | 0.93 |
| Used ANC | 1.27 | 0.78 |
| Place of delivery | 1.51 | 0.66 |
| Insurance | 1.09 | 0.92 |
| Distance to a health facility | 1.12 | 0.89 |
| Wealth status | 1.78 | 0.56 |
| Currently working | 1.05 | 0.95 |
| Country | 1.21 | 0.82 |

**Note:** VIF - variance inflation factor. TI - tolerance indices
