# Supplementary material for: Factors associated with weighing a child at birth: Evidence from 16 sub-Saharan African countries
Source: PLoS One. 2025 Jul 22;20(7):e0328463. doi: 10.1371/journal.pone.0328463 (PMC12282927; doi:10.1371/journal.pone.0328463)
Supplement: Attachment — Submitted filename: PONE-D-24-00704_R3-AF.pdf [file pone.0328463.s009.pdf]

PLOS ONE

Factors associated with weighing a child at birth: evidence from 16 sub-Saharan African countries

--Manuscript Draft--

**Manuscript Number:**	PONE-D-24-00704R3
**Article Type:**	Research Article
**Full Title:**	Factors associated with weighing a child at birth: evidence from 16 sub-Saharan African countries
**Short Title:**	Weighing a child at birth: evidence from 16 sub-Saharan African countries
**Corresponding Author:**	Edward Nketiah-Amponsah, PhD<br>University of Ghana<br>Accra, GHANA
**Keywords:**	child; birthweight; Weighed at birth; Logistic regression; sub-Saharan Africa
**Abstract:**	Background: Several children from sub-Saharan Africa (SSA) are not weighed at birth. The lack of birthweight data is a significant challenge in monitoring the global prevalence of extreme birthweight, either low or high, and newborn health. This data guides resource allocation and the design of targeted health policies to address neonatal complications and mortalities. This paper explores the demand-side predictors of newborn weighing.<br>Methods: Data were obtained from the Demographic and Health Surveys (DHS) of 16 countries in SSA, conducted from 2014 to 2021. Multivariate logistic regression was used to achieve the study’s objectives.<br>Results: Approximately 59% of the study population were weighed at birth. This prevalence rate varied widely across the 16 countries, ranging from 23% in Chad to 94% in Gabon. The study documents a positive association between higher socioeconomic status and the probability of being weighed at birth. Specifically, older women and women with higher education and wealth were more likely to weigh their newborns at birth. Also, women who delivered at healthcare facilities and those who used antenatal care had a higher likelihood of weighing their children at birth. Urban residents were more likely to weigh their children at birth. On the contrary, the likelihood of weighing a child at birth decreases with parity.<br>Conclusion: The study highlights the need to target pregnant women of lower socioeconomic status for interventions aimed at averting severe morbidity and mortality occasioned by conditions of low birthweight.
**Order of Authors:**	Alex Bawuah
	Samuel Ampaw, PhD
	Edward Nketiah-Amponsah, PhD
**Opposed Reviewers:**	
**Response to Reviewers:**	We sincerely thank you for your constructive editorial comments on our manuscript titled ‘Factors associated with weighing a child at birth: evidence from 16 sub-Saharan African countries’ submitted to PLOS ONE for publication consideration. The comments have been invaluable in improving the quality of our manuscript.<br>In particular, we requested a colleague who is more proficient in the English language to proofread the manuscript. We have also diligently addressed all the minor but essential errors, such as omitting the percent (%) sign in some figures. Besides, we have accepted your suggested corrections in tracked changes across the manuscript. For ease of reference, we have provided the clean and tracked changes versions of the manuscript for your review.<br><br>We hope our revisions will meet your kind expectations.
**Additional Information:**	
**Question**	**Response**
**Financial Disclosure**	The author(s) received no specific funding for this work.

Powered by Editorial Manager® and ProduXion Manager® from Aries Systems Corporation

Enter a financial disclosure statement that describes the sources of funding for the work included in this submission. Review the submission guidelines for detailed requirements. View published research articles from *PLOS ONE* for specific examples.<br><br>This statement is required for submission and **will appear in the published article** if the submission is accepted. Please make sure it is accurate.<br><br>**Funded studies**<br>Enter a statement with the following details:<br>• Initials of the authors who received each award<br>• Grant numbers awarded to each author<br>• The full name of each funder<br>• URL of each funder website<br>• Did the sponsors or funders play any role in the study design, data collection and analysis, decision to publish, or preparation of the manuscript?<br><br>Did you receive funding for this work?	
**Competing Interests**<br><br>Use the instructions below to enter a competing interest statement for this submission. On behalf of all authors, disclose any competing interests that could be perceived to bias this work—acknowledging all financial support and any other relevant financial or non-financial competing interests.<br><br>This statement is required for submission and **will appear in the published article** if the submission is accepted. Please make sure it is accurate and that any funding sources listed in your Funding Information later in the submission form are also declared in your Financial Disclosure statement.	I have read the journal's policy and the authors of this manuscript have the following competing interests:<br>no competing interest.

View published research articles from *PLOS ONE* for specific examples.<br><br>**NO authors have competing interests**<br><br>Enter: *The authors have declared that no competing interests exist.*<br><br>**Authors with competing interests**<br><br>Enter competing interest details beginning with this statement:<br><br>*I have read the journal's policy and the authors of this manuscript have the following competing interests: [insert competing interests here]*<br><br>* typeset	
**Ethics Statement**<br><br>Enter an ethics statement for this submission. This statement is required if the study involved:<br><br>• Human participants<br>• Human specimens or tissue<br>• Vertebrate animals or cephalopods<br>• Vertebrate embryos or tissues<br>• Field research<br><br>Write "N/A" if the submission does not require an ethics statement.<br><br>General guidance is provided below. Consult the submission guidelines for detailed instructions. **Make sure that all information entered here is included in the Methods section of the manuscript.**	N/A

**Format for specific study types**<br><br>**Human Subject Research (involving human participants and/or tissue)**<br>• Give the name of the institutional review board or ethics committee that approved the study<br>• Include the approval number and/or a statement indicating approval of this research<br>• Indicate the form of consent obtained (written/oral) or the reason that consent was not obtained (e.g. the data were analyzed anonymously)<br><br>**Animal Research (involving vertebrate animals, embryos or tissues)**<br>• Provide the name of the Institutional Animal Care and Use Committee (IACUC) or other relevant ethics board that reviewed the study protocol, and indicate whether they approved this research or granted a formal waiver of ethical approval<br>• Include an approval number if one was obtained<br>• If the study involved *non-human primates*, add *additional details* about animal welfare and steps taken to ameliorate suffering<br>• If anesthesia, euthanasia, or any kind of animal sacrifice is part of the study, include briefly which substances and/or methods were applied<br><br>**Field Research**<br><br>Include the following details if this study involves the collection of plant, animal, or other materials from a natural setting:<br>• Field permit number<br>• Name of the institution or relevant body that granted permission	
**Data Availability**<br><br>Authors are required to make all data underlying the findings described fully available, without restriction, and from the time of publication. PLOS allows rare exceptions to address legal and ethical concerns. See the PLOS Data Policy and FAQ for detailed information.	Yes - all data are fully available without restriction

A Data Availability Statement describing where the data can be found is required at submission. Your answers to this question constitute the Data Availability Statement and **will be published in the article**, if accepted.<br><br>**Important:** Stating 'data available on request from the author' is not sufficient. If your data are only available upon request, select 'No' for the first question and explain your exceptional situation in the text box.<br><br>Do the authors confirm that all data underlying the findings described in their manuscript are fully available without restriction?	
**Describe where the data may be found in full sentences. If you are copying our sample text, replace any instances of XXX with the appropriate details.**<br><br>• If the data are **held or will be held in a public repository**, include URLs, accession numbers or DOIs. If this information will only be available after acceptance, indicate this by ticking the box below. For example: *All XXX files are available from the XXX database (accession number(s) XXX, XXX.).*<br>• If the data are all contained **within the manuscript and/or Supporting Information files**, enter the following: *All relevant data are within the manuscript and its Supporting Information files.*<br>• If neither of these applies but you are able to provide **details of access elsewhere**, with or without limitations, please do so. For example:<br><br>*Data cannot be shared publicly because of [XXX]. Data are available from the XXX Institutional Data Access / Ethics Committee (contact via XXX) for researchers who meet the criteria for access to confidential data.*<br><br>*The data underlying the results presented in the study are available from (include the name of the third party*	The data is owned by a third party – Demographic and Health Survey (DHS). The data is publicly available on the DHS website (www.dhsprogram.com). The data specific to our empirical analysis in this paper has been shared as supporting information

and contact information or URL).<br>• This text is appropriate if the data are owned by a third party and authors do not have permission to share the data.<br><br>* typeset	
Additional data availability information:	Tick here if the URLs/accession numbers/DOIs will be available only after acceptance of the manuscript for publication so that we can ensure their inclusion before publication.

Powered by Editorial Manager® and ProduXion Manager® from Aries Systems Corporation

DEPARTMENT OF ECONOMICS

UNIVERSITY OF GHANA

Telephone: 501485, 500381
Telegraph & CABLES – UNIVERSITY, LEGON
Telex: 2556 UGL.GH
Fax: 233-021-501486
Email: economics@ug.edu.gh

P. O. BOX LG57
LEGON, ACCRA
GHANA

June 8, 2025

Dear Editor,

<u>SUBMISSION OF REVISED MANUSCRIPT FOR PUBLICATION IN PLOS ONE</u>

We sincerely thank you for your constructive editorial comments on our manuscript titled '**Factors associated with weighing a child at birth: evidence from 16 sub-Saharan African countries'** submitted to PLOS ONE for publication consideration. The comments have been invaluable in improving the quality of our manuscript.

In particular, we requested a colleague who is more proficient in the English language to proofread the manuscript. We have also diligently addressed all the minor but essential errors, such as omitting the percent (%) sign in some figures. Besides, we have accepted your suggested corrections in tracked changes across the manuscript. For ease of reference, we have provided the clean and tracked changes versions of the manuscript for your review.

We hope our revisions will meet your kind expectations.

Yours faithfully,

Prof. Edward Nketiah-Amponsah, PhD (on behalf of all co-authors)

Revised Manuscript (Clean)

Click here to access/download;Manuscript;Revised Manuscript _Clean_June 11 2025.docx

Factors associated with weighing a child at birth: evidence from 16 sub-Saharan African countries

Alex Bawuah[1], ORCID: 0000-0003-4603-5789

Samuel Ampaw[2], ORCID: 0000-0001-5881-4347

Edward Nketiah-Amponsah[3], ORCID: 0000-0002-9754-1983

[1]Health Economics Research Unit, Aberystwyth Business School, Aberystwyth University, Aberystwyth, Ceredigion, Wales, UK, SY23 3DY.

[2]Global Poverty Research Lab, Kellogg School of Management, Northwestern University, Evanston, Illinois, USA.

[*3]Department of Economics, University of Ghana, Legon.

enamponsah@ug.edu.gh

Abstract

Background: Several children from sub-Saharan Africa (SSA) are not weighed at birth. The lack of birthweight data is a significant challenge in monitoring the global prevalence of extreme birthweight, either low or high, and newborn health. This data guides resource allocation and the design of targeted health policies to address neonatal complications and mortalities. This paper explores the demand-side predictors of newborn weighing.

Methods: Data were obtained from the Demographic and Health Surveys (DHS) of 16 countries in SSA, conducted from 2014 to 2021. Multivariate logistic regression was used to achieve the study's objectives.

Results: Approximately 59% of the study population were weighed at birth. This prevalence rate varied widely across the 16 countries, ranging from 23% in Chad to 94% in Gabon. The study documents a positive association between higher socioeconomic status and the probability of being weighed at birth. Specifically, older women and women with higher education and wealth were more likely to weigh their newborns at birth. Also, women who delivered at healthcare facilities and those who used antenatal care had a higher likelihood of weighing their children at birth. Urban residents were more likely to weigh their children at birth. On the contrary, the likelihood of weighing a child at birth decreases with parity.

Conclusion: The study highlights the need to target pregnant women of lower socioeconomic status for interventions aimed at averting severe morbidity and mortality occasioned by conditions of low birthweight.

Keywords: Child; Birthweight; Weighed at birth; sub-Saharan Africa.

Background

Birthweight is the first weight of the foetus or newborn measured after birth [1]. It can be categorized into three: low birthweight (birthweight ≤ 2.5 kg), normal birthweight (birthweight between 2.5kg – 4.0kg), and macrosomia or high birthweight (birthweight > 4.0 kg) [2,3]. A child's birthweight is an essential indicator of their health and development, as it can affect their short- and long-term outcomes [4]. For instance, low birthweight babies are at a higher risk of developing chronic diseases [5], stunted growth [6], lower IQ [7], and reduced life expectancy [8]. High birthweight is associated with high risks for certain malignancies in childhood, hypertension in childhood, psychiatric disorders, type 1 and 2 diabetes, breast cancer, and obesity [9–11].

Given the risk factors associated with high and low birthweight, the WHO recommends that babies be weighed during postnatal care [12]. Weighing children at birth has several merits. First, knowledge about the birthweight provides vital information about a child's health [5]. It guides medical care and interventions, such as medications and feeding plans, to promote the best possible outcomes for the child's health and development. For instance, babies with low or high birthweight may need to be fed differently or be given particular medications compared to babies with normal birthweight. Second, weighing newborns helps monitor the prevalence of low or high birthweight [8]. Third, a child's birthweight can give important information about the mother's health. Studies have shown that mothers who have poor nutrition, anaemic conditions, and are underweight are more likely to have low birthweight babies [13,14]. Fourth, studies relating to a child's birthweight have often been centred around the factors that influence a child's birthweight [15–20]. Researchers were able to conduct these studies because the children were weighed at birth and their birthweights recorded. These studies could not have been undertaken if the children had not been weighed at birth.

The challenge, however, is that many newborns are not weighed at birth. For instance, in 2015, nearly one-third of newborns (39.7 million) globally were not weighed at birth, with Africa recording the highest percentage of newborns without a recorded birthweight (51.7%, or 21.5 million) [8]. This situation places a significant challenge on monitoring the prevalence of low or high birthweight and the health of newborns due to the lack of birthweight data for many children. This study, therefore, aims to investigate the factors associated with weighing a child at birth in sub-Saharan Africa (SSA).

Various factors, including individual sociodemographic, maternal, household, and community-level factors, can influence the decision to weigh a child at birth. Understanding these factors can guide policymakers in implementing policies that will encourage the weighing of newborns. To the best of our knowledge, there has been no published literature on this topic. This study is the first to explore the factors influencing the decision to weigh newborns.

Methods

Data source

The study used data from the Demographic and Health Surveys (DHS) of 16 countries in SSA conducted from 2014 to 2021 (these are countries with high neonatal mortality rates for which data were available). The DHS is a nationwide survey conducted in over 85 low- and middle-income countries worldwide and follows a consistent protocol and terminology across all countries [21]. It employs a structured questionnaire to gather information on various health indicators, including maternal and child health, fertility, family planning utilization, morbidity, and mortality [21]. The DHS uses a two-stage sampling technique to collect data, starting with selecting enumeration areas based on each country's sampling frame, followed by the selection of households from each enumeration area. Detailed information on the sampling and data collection methods can be found in the work of Aliaga and Ren [22].

This study employed the children's dataset (KR file) from the DHS surveys. The KR file contains a single entry for each child born to the interviewed women within the five years preceding the survey [23]. The total sample size of children (aged 0 to 5 years) from the sixteen countries in the specified duration was 183,541.

Variables

The study's outcome variable is whether the child was weighed at birth. In the survey, the respondents were asked if the child was weighed at birth. The response was coded as "1 = Yes" and "0 = No" for children who were weighed and those who were not weighed at birth, respectively.

The study used a total of 13 explanatory variables. The variables include the respondent's age, woman's highest level of education, marital status, residence, parity, decisions about woman's health, used antenatal care (ANC), place of delivery, covered by insurance, household wealth status, birth type, sex of the child, currently working status and distance to a healthcare facility. Respondent's age was coded on a five-interval scale from 15 to 49. Women's education was

coded as "none", "primary", "secondary", and "higher". Marital status was coded as "married" and "otherwise". The residence was coded as "rural" and "urban". Parity indicates the number of times the woman has had a live birth. Decisions about women's health were coded as "woman alone", "together with partner/husband", and "husband/partner alone". ANC use was coded as "yes" and "no". The place of delivery (childbirth) was coded as "health facility" and "otherwise". Covered by insurance was coded as "yes" and "no". Household wealth status was coded as "poorest", "poorer", "middle", "richer", and "richest". The birth type was coded as "single birth" and "multiple births". Child sex was coded as "male" and "female". Distance to healthcare facilities was coded as "a big problem" and "not a big problem" (in the survey, the women were asked whether distance to the healthcare facility was a big problem or not a big problem).

The year variable (the year in which the survey was conducted) was initially included in the model to account for potential temporal effects. However, we found that it was perfectly collinear with the country variable. Given this collinearity, the year variable was omitted from the final model. Furthermore, the model did not include birth order due to collinearity with the parity variable.

Data analysis

The data were analysed using STATA version 16. Frequency tables were used to describe the data (Table 2). Given the binary nature of the dependent variable, the study applied logistic regressions to evaluate the determinants of measuring a child's weight at birth [24]. First, a bivariate logistic regression analysis was conducted to estimate the unadjusted odds ratios (ORs) for each determinant. Afterwards, a multivariable logistic regression was performed to estimate the adjusted odds ratios (AORs) for all determinants, considering clustering (at the country level) and sampling weights. We handled missing data using listwise deletion; thus, observations with missing values in any of the variables included in the regression model were excluded from the analysis. This approach ensures consistency across all model estimates.

Prior to the regression analysis, we conducted a diagnostic test for collinearity between the independent variables by looking at the variance inflation factor (VIF) and tolerance indices (TI) of the independent variables. VIF and TI are measures used to assess the level of multicollinearity between the *i*th independent variable and other independent variables in a regression model [25]. VIFs above 5 and TIs less than 0.20 are considered signs of multi-

collinearity [26] . The VIFs for all the independent variables used in the study were below 5. Similarly, the TIs were above 0.20. These results are provided in Table A1 in the appendix.

Ethical Consideration

The data used for this study, Demographic and Health Survey (DHS) is secondary, and its usage does not require ethical clearance. Measure DHS, which is responsible for undertaking standardized Demographic and Health Surveys in over 90 countries, duly obtained ethical clearance from the requisite institutions in all 16 countries included in this study, as well as the Institutional Review Board of the ICF International. In this regard, ethical approval and consent of participants to participate in the study are not applicable. It is worth noting that all data are anonymous and confidential, so there is no way of identifying individuals in the database.

Results

Descriptive statistics

As shown in Table 1, the sample size for the study was 183,541. Almost 60% of the children were weighed at birth. Rwanda had the highest percentage of children weighed at birth (95.12%), whereas Ethiopia had the lowest (27.48%). Most of the countries recorded a less than 70% prevalence of babies weighed at birth (Angola – 55.14%; Cameroon – 69.19%; Ethiopia – 27.48%; Gambia 63.81%; Guinea – 51.06%; Kenya – 59.51%; Liberia – 65.83%; Madagascar – 38.53%; Malawi – 52.66%; Nigeria – 29.28%).

Table 1 – Sample size characteristics

Country	Year of survey	Number of children	% of Children weighed at birth
Angola	2015-2016	14322	55.14
Benin	2017-2018	13589	76.48
Cameroon	2018	9733	69.19
Ethiopia	2016	10641	27.48
Gambia	2019-2020	5447	63.81
Ghana	2014	8362	86.04
Guinea	2018	7951	51.06
Kenya	2014	9967	59.51
Liberia	2019-2020	5704	65.83
Madagascar	2021	12499	38.53
Malawi	2015-2016	9940	52.66
Mali	2018	17286	91.7

Nigeria	2018	33924	29.28
Rwanda	2019-2020	8092	95.12
Senegal	2019	6125	76.91
Zambia	2018	9959	83.2
Total		183541	59.33

Table 2 presents the results of the descriptive summary of the sample. It shows that most of the children are males (50.73%). Also, a higher proportion of the respondents are between 25 and 29 years old (26.79%). Likewise, most of the respondents were married (74.62%), rural residents (67.99%), had a single birth (96.47%), used ANC (87.16%), and reported that distance to a healthcare facility was not a big problem (61.02%). Most of the respondents did not have insurance (91.5%).

Furthermore, most of the children who were weighed at birth were delivered in a healthcare facility (91.71%), whereas those who were not weighed were delivered elsewhere (83.18%), mostly at home (81.74%). Similarly, a higher proportion of those who weighed their children had primary education (36.61%), whereas those who did not weigh their children had no education (59.18%). Likewise, a higher proportion of those who weighed their children belonged to the wealthiest wealth category (21.6%), whereas those who did not weigh their children belonged to the poorest category (39.95%). Also, the mean parity of those who did not weigh their children was higher (4.45) than those who weighed their children (3.53).

Table 2 – Descriptive statistics of the sample

Variable	Pooled Sample Frequency (%)	Weighed Frequency (%)	Not Weighed Frequency (%)	χ^2/ Diff.t-test
**Child's Characteristics**				
Weighed at birth (*n* =183541)				
Yes	108892 (59.33)			
No	74649 (40.67)			
Missing = 0 (0.00%)				
Child's sex (*n* =183541)				
Male	93104 (50.73)	55527 (50.99)	37577 (50.34)	0.006
Female	90437 (49.27)	53365 (49.01)	37072 (49.66)	
Missing = 0 (0.00%)				
Birth type (*n* =183541)				
Single birth	177062 (96.47)	104721 (96.17)	72350 (96.92)	<0.001
Multiple births	6473 (3.53)	4173 (3.83)	2301 (3.08)	
Missing = 0 (0.00%)				
**Mother's Characteristics**				
Age (*n* =183541)				
15 – 19	11510 (6.27)	6733 (6.18)	4777 (6.40)	<0.001
20 – 24	41277 (22.49)	24796 (22.77)	16481 (22.08)	
25 – 29	49173 (26.79)	29188 (26.80)	19985 (26.77)	

30 – 34	38206 (20.82)	23069 (21.19)	15137 (20.28)	
35 – 39	27072 (14.75)	16233 (14.91)	10839 (14.52)	
40 – 44	12267 (6.68)	6885 (6.32)	5382 (7.21)	
45 – 49	4036 (2.20)	1988 (1.83)	2048 (2.74)	
Missing = 0 (0.00%)				
Level of education (*n* =183539)				
None	73968 (40.30)	29790 (27.36)	44178 (59.18)	<0.001
Primary	59385 (32.36)	39865 (36.61)	19520 (26.15)	
Secondary	43334 (23.61)	33059 (30.36)	10277 (13.77)	
Higher	6853 (3.73)	6179 (5.68)	674 (0.90)	
Missing = 2 (0.00%)				
Marital Status (*n* =183541)				
Married	136961 (74.62)	78307 (71.91)	58654 (78.57)	<0.001
Otherwise	46580 (25.38)	30585 (28.09)	15995 (21.43)	
Missing = 0 (0.00%)				
Residence (*n* =183541)				
Urban	58753 (32.01)	45620 (41.89)	13123 (17.58)	<0.001
Rural	124798 (67.99)	63272 (58.11)	61526 (82.42)	
Missing = 0 (0.00%)				
Parity (*n* =183541)	*Mean* = 3.91	*Mean* = 3.53	*Mean* = 4.45	<0.001
Missing = 0 (0.00%)				
Health decisions (*n* =159575)				
Alone	26163 (16.40)	17312 (18.67)	8851 (13.24)	<0.001
Together with partner/husband	63640 (39.88)	39815 (42.94)	23825 (35.64)	
Husband/partner alone	69772 (43.72)	35600 (38.39)	34172 (51.12)	
Missing = 23966 (13.06%)				
Used ANC (*n* =125712)				
Yes	109568 (87.16)	77486 (97.95)	32082 (68.84)	<0.001
No	16144 (12.84)	1625 (2.05)	14519 (31.16)	
Missing = 57829 (31.51%)				
Place of delivery (*n* =183530)				
Health facility	112413 (61.25)	99857 (91.71)	12556 (16.82)	<0.001
Otherwise	71117 (38.75)	9028 (8.29)	62089 (83.18)	
Missing = 11 (0.01%)				
Insurance (*n* =177413)				
Yes	15077 (8.50)	12689 (12.18)	2388 (3.26)	<0.001
No	162336 (91.50)	91489 (87.82)	70847 (96.74)	
Missing = 6128 (3.34%)				
Distance to a facility (*n* =183535)				
A big problem	71538 (38.98)	35623 (32.72)	35915 (48.11)	<0.001
Not a big problem	111997 (61.02)	73265 (67.28)	38732 (51.89)	
Missing = 6 (0.00%)				
Currently working (*n* =183,525)				
Yes	117785 (64.18)	71339 (65.52)	46446 (62.22)	
No	65740 (35.82)	37540 (34.48)	28200 (37.78)	<0.001
Currently working (*n* =183,525)				
**Household Characteristics**				
Wealth status (*n* =183541)				
Poorest	48380 (26.36)	18561 (17.05)	29819 (39.95)	<0.001
Poorer	41210 (22.45)	20871 (19.17)	20339 (27.25)	
Middle	36739 (20.02)	22903 (21.03)	13836 (18.53)	
Wealthier	30732 (16.74)	23032 (21.15)	7700 (10.31)	
Wealthiest	26480 (14.43)	23525 (21.60)	2955 (3.96)	
Missing = 0 (0.00%)				

Determinants of weighing children at birth

Table 3 presents the findings from both the bivariate and multivariable logistic regression analyses. Results of the multivariable logistic regression show that age, parity, place of delivery, residence, education, wealth status, the person(s) who decides on the woman's healthcare, ANC utilisation, distance to a health facility, and having insurance were significant determinants of weighing children at birth. The odds of weighing children at birth for respondents aged between 20 – 24, 25 – 29, 30 – 34, 35 – 39, 40 – 44, and 45 – 49 were respectively 1.14 (95% CI=1.01 - 1.29), 1.30 (95% CI=1.03 - 1.63), 1.57 (95% CI=1.17 - 2.09), 1.85 (95% CI=1.35 - 2.54), 1.9[illegible]5% CI=1.32 - 2.76), and 1.67 (95% CI=1.14 - 2.44) times higher than those between the ages of 15 – 19. Furthermore, relative to the uneducated, the odds of weighing children at birth were 1.38 (95% CI=1.19 - 1.60), 1.64 (95% CI=1.41 - 1.91), and 2.68 (95% CI=2.33 - 3.08) times higher for women with primary, secondary, and higher education, respectively. Similarly, the probability of weighing children at birth was 1.20 (95% CI=1.07 - 1.35), 1.48 (95% CI=1.22 - 1.78), 2.05 (95% CI=1.56 - 2.70), and 3.91 (95% CI=2.96 - 5.17) times higher for those from poorer, middle, wealthier and wealthiest households, respectively, compared to those from the poorest households. Women who delivered at healthcare facilities were more likely to weigh their children at birth (AOR=52.29, 95% CI=38.43 - 71.16). Similarly, women who used ANC during pregnancy were more likely to weigh their children at birth (AOR=4.62, 95% CI=3.76 - 5.67). Urban residents were more likely to weigh their children at birth (AOR=2.29, 95% CI=1.79 - 2.91) than rural residents. Also, the insured are more likely to weigh their children at birth (AOR=1.32, 95% CI=1.09 - 1.61). In addition, women who make decisions about their healthcare alone and those who decide jointly with their husbands/partners are 1.28 (95% CI=1.04 - 1.57) and 1.22 (95% CI=1.01 - 1.46) times more likely than women whose husbands/partners alone make decisions about their healthcare. Furthermore, those who reported that distance to the healthcare facility is not a big problem were more likely to weigh their children (AOR=1.09, 95% CI=1.00 - 1.19) than their counterparts who had difficulty accessing the healthcare facility. Moreover, the likelihood of weighing children at birth decreases as parity increases (AOR=0.94, 95% CI=0.90 - 0.98). Furthermore, children from other countries are less likely to be weighed compared to children from Rwanda.

The interactions between residence and place of birth showed that urban residents who give birth in healthcare facilities are 54.84 times more likely to weigh their children compared to rural residents who give birth at other places. Similarly, women who used ANC services and

gave birth in a healthcare facility were 166.69 times more likely to weigh their children than those who did not use ANC and gave birth elsewhere. Also, compared to rural residents with no education, urban residents with secondary levels of education and urban residents with higher levels of education are 5.12 and 8.80 (respectively) times more likely to weigh their children.

Table 3 – Determinants of weighing children at birth in sub-Saharan Africa

Variables	Unadjusted OR	Adjusted OR
**Child's Characteristics**		
Child sex (Ref: Male)		
Female	0.97 (0.96 - 1.00)**	1.02 (0.97 - 1.07)
Birth type (Ref: Single)		
Multiple	1.21 (1.03 - 1.41)**	1.03 (0.89 - 1.20)
**Mother's Characteristics**		
Age at birth (Ref: 15 – 19)		
20 – 24	1.10 (0.95 - 1.28)	1.14 (1.01 - 1.29)**
25 – 29	1.08 (0.83 - 1.40)	1.30 (1.03 - 1.63)**
30 – 34	1.13 (0.84 - 1.50)	1.57 (1.17 - 2.09)***
35 – 39	1.11 (0.80 - 1.55)	1.85 (1.35 - 2.54)***
40 – 44	0.94 (0.69 - 1.27)	1.91 (1.32 - 2.76)***
45 – 49	0.68 (0.52 - 0.90)***	1.67 (1.14 - 2.44)***
Education level (Ref: None)		
Primary	3.11 (1.69 - 5.72)***	1.38 (1.19 - 1.60)***
Secondary	5.19 (3.33 - 8.07)***	1.64 (1.41 - 1.91)***
Higher	15.16 (8.70 - 26.41)***	2.68 (2.33 - 3.08)***
Marital Status (Ref: Otherwise)		
Married	0.64 (0.36 - 1.13)	1.15 (0.84 - 1.57)
Residence (Ref: Rural)		
Urban	3.78 (2.21 - 6.47)***	2.29 (1.79 - 2.91)***
Parity	0.84 (0.81 - 0.87)***	0.94 (0.90 - 0.98)***
Health decisions (Ref: Husband/partner alone)		
Woman Alone	1.97 (1.02 - 3.82)**	1.28 (1.04 - 1.57) **
Together with partner/husband	1.64 (0.81 - 3.32)	1.22 (1.01 - 1.46) **
Used ANC (Ref: No)		
Yes	21.76 (16.25 - 29.15)***	4.61 (3.76 - 5.67)***
Place of delivery (Ref: Otherwise)		
Health facility	54.60 (34.47 - 86.48)***	52.29 (38.43 - 71.16)***
Insurance (Ref: No)		
Yes	4.35 (1.48 - 12.85)***	1.32 (1.09 - 1.61)***
Distance to health facility (Ref: big problem)		
Not a big problem	1.79 (1.14 - 2.83)**	1.09 (1.00 - 1.19)*
Currently working (Ref: No)		
Yes	1.19 (0.77 - 1.83)	1.01 (0.94 - 1.09)
**Household Characteristics**		
Wealth status (Ref: Poorest)		
Poorer	1.51 (1.24 - 1.84)***	1.20 (1.07 - 1.35)***

Middle	2.40 (1.67 - 3.43)***	1.48 (1.22 - 1.78)***
Wealthier	4.52 (2.85 - 7.16)***	2.05 (1.56 - 2.70)***
Wealthiest	12.77 (7.89 - 20.67)***	3.91 (2.96 - 5.17)***
**Countries** (Ref: Rwanda)		
Angola	0.08 (0.08 - 0.08)***	0.43 (0.35 - 0.53)***
Benin	0.17 (0.17 - 0.17)***	0.21 (0.16 - 0.28)***
Cameroon	0.11 (0.11 - 0.11)***	0.23 (0.19 - 0.27)***
Ethiopia	0.01 (0.01 - 0.01)***	0.05 (0.04 - 0.06)***
Ghana	0.10 (0.10 - 0.10)***	0.10 (0.09 - 0.11)***
Gambia	0.49 (0.49 - 0.49)***	0.87 (0.70 - 1.09)
Guinea	0.06 (0.06 - 0.06)***	0.19 (0.15 - 0.24)***
Kenya	0.10 (0.10 - 0.10)***	0.22 (0.19 - 0.26)***
Liberia	0.11 (0.11 - 0.11)***	0.08 (0.06 - 0.10)***
Madagascar	0.03 (0.03 - 0.03)***	0.09 (0.08 - 0.11)***
Mali	0.06 (0.06 - 0.06)***	0.10 (0.07 - 0.14)***
Malawi	0.53 (0.53 - 0.53)***	0.57 (0.46 - 0.71)***
Nigeria	0.02 (0.02 - 0.02)***	0.03 (0.02 - 0.04)***
Senegal	0.21 (0.21 - 0.21)***	0.35 (0.27 - 0.46)***
Zambia	0.29 (0.29 - 0.29)***	0.35 (0.28 - 0.44)***
**Interactions**		
Residence & Birthplace (Ref: Rural & Otherwise)		
Urban*Health facility		0.46 (0.36 - 0.59)***
ANC & Birthplace (Ref: No ANC & Otherwise)		
Used ANC* Health facility		0.69 (0.54 - 0.87)***
Residence & Education ((Ref: Rural & No education)		
Urban*Primary		1.13 (0.93 - 1.38)
Urban*Secondary		1.37 (1.11 - 1.70)***
Urban*Higher		1.44 (1.07 - 1.94)**
Constant		0.13 (0.10 - 0.17)***
Log pseudolikelihood		-2.821e+10
Pseudo R^2		0.59
Observations		102,709

Note: OR = odds ratio; Ref = Reference group. Sampling weights and clustering (at country level) accounted for in the regression analysis. 95% confidence interval (CI) in parentheses. *** p<0.01, ** p<0.05, * p<0.10

Discussion

The study examined the factors associated with weighing a child at birth using the most recent DHS data from 16 SSA countries. We found that older women were more likely to weigh their children than younger women. Studies have found that older women have a higher chance of encountering complications during pregnancy, which could have an adverse effect on their

children [25,26]. Knowing this information, older women may want to weigh their children to safeguard their health.

Educated women were more likely to weigh their children than uneducated women. This finding may be because the educated are better informed about the benefits of weighing children at birth than the uneducated. For instance, women with higher levels of education may learn about the importance of weighing children at birth. Moreover, studies have documented the correlation between education and health outcomes for women and children [27–29].

Parity was negatively correlated with the odds of weighing a child. The study revealed that the likelihood of weighing a child at birth decreases as parity increases. This finding may be linked with the observation that women with more pregnancy and birthing experience can often produce healthy babies [30,31]. Therefore, women with more parity may not see the need to weigh their children.

The results from the study further revealed that women covered by health insurance are more likely to weigh their children at birth. Health insurance may cover the cost of healthcare services, which include weighing a child, hence encouraging women, especially those with low incomes, to weigh their children. Moreover, insurance increases access to healthcare services [32–34]; hence, women with insurance may be more likely to use healthcare services such as weighing their children at birth.

The study further revealed a positive association between wealth status and weighing children at birth. The likelihood of weighing a child at birth increases with household wealth. One possible explanation behind this finding could be the cost of transportation to healthcare facilities. The poor may be unable to afford the cost of transporting their babies to healthcare facilities to have their weight checked, unlike the wealthy, who can afford it. Another possible explanation may be that low-income families prioritize meeting their fundamental daily necessities, resulting in fewer resources for healthcare (such as weighing their babies) compared to affluent households. This finding (the positive correlation between wealth and weighing of children at birth) suggests implementing anti-poverty measures to encourage weighing children at birth.

The use of ANC was also positively correlated with the weighing of children at birth. This finding may be linked to the components of ANC, which include health education and promotion, prevention and management of pregnancy-related or concurrent diseases, and risk detection [35]. Thus, women who use ANC are educated or informed about the importance of

weighing children at birth, perhaps explaining why they have a higher chance of weighing their children.

Place of residence also emerged as a significant determinant of weighing children at birth. The study found that rural residents are less likely to weigh their children. Individuals who live in urban areas have better access to a range of healthcare facilities and healthcare personnel, which makes it easier for them to access healthcare services [36], including the weighing of their children.

Women who gave birth at healthcare facilities were more likely to weigh their children at birth. There are two possible explanations for this finding. The first relates to the availability of resources at the healthcare facilities, such as the tools/equipment used to weigh children and the personnel who can weigh the children. The second could be linked to the fact that women who deliver at healthcare facilities are more likely to receive postnatal care, which also includes weighing children [37,38].

One of the strengths of this study is that it is the first to investigate the factors that influence the decision to weigh a child at birth. This is a significant contribution to the literature, given that there has been no published literature on this topic. Another strength of this study is that it used nationally representative data from 16 sub-Saharan African countries to examine the factors influencing the decision to weigh a child at birth. Therefore, lessons from this study can be relevant to other countries in sub-Saharan Africa (due to the similarity in the healthcare systems and population characteristics among sub-Saharan African countries).

One limitation of this study is that potential supply-side predictors such as the healthcare facility, personnel, and weighing equipment availability were not accounted for due to data limitations. Future studies can explore the extent to which these supply-side variables influence the decision to weigh a newborn. Another limitation is that the study only establishes association, not causality. Nonetheless, the study offers a foundation for more rigorous analysis in future studies.

Conclusion

The study investigated the factors associated with weighing a child at birth using nationally representative data from 16 SSA countries. The overall prevalence of weighing children at birth was 59%. This ranged from 27.48% in Ethiopia to 95.12% in Rwanda. Results from the study's multivariable regression showed that ~~old~~ age, having an education, being married, giving birth

in a healthcare facility, having insurance, using ANC, living in urban areas, having autonomy in making healthcare decisions and having a higher wealth status were positively correlated (significantly) with weighing children at birth. However, higher parity was negatively correlated (significantly) with weighing children at birth.

The study's findings have some important policy implications. The finding that health facility delivery and using ANC positively correlate with weighing a child at birth suggests implementing policies/programs to encourage ANC use and facility delivery. One such policy could be creating awareness programs to educate women of childbearing age about the importance of using ANC and facility delivery. This can be done through local media, social media, and other media platforms. Furthermore, the study also provides support for women empowerment programmes. The statistical significance of autonomy in healthcare decisions and formal education suggests the need to institute policies promoting girl child education, such as scholarships for brilliant but needy girls. Moreover, the literature highlights the positive correlation between education and the autonomy of women in decision-making [39–41]. Special considerations could be given to the poor and rural residents in adopting these interventions.

List of abbreviations

OR: Odds Ratio

AOR: Adjusted Odds Ratio

CI: Confidence Interval

ANC: Antenatal Care

SSA: Sub-Sahara Africa

DHS: Demographic and Health Survey

WHO: World Health Organisation

IQ: Intelligence Quotient

VIF: Variance Inflation Factor

TI: Tolerance Indices

Declarations

Acknowledgement

We thank the Demographic and Health Survey (DHS) program for granting us access to the DHS data for the 16 countries.

Authors' Contributions

AB: Conceptualisation, Methodology, Validation, Formal Analysis, Investigation, Resources, Data Curation, Writing—Original Draft, Writing---Review & Editing, Visualisation, Project Administration.

SA: Methodology, Validation, Formal Analysis, Investigation, Resources, Data Curation, Writing---Review & Editing, Visualisation.

ENA: Conceptualisation, Methodology, Formal Analysis, Validation, Resources, Writing---Review & Editing, Visualisation.

Funding

No funding was received for conducting this study.

Availability of Data

The data is owned by a third party – Demographic and Health Survey (DHS). The data are publicly available on the DHS website (www.dhsprogram.com). Interested parties can request for the data from DHS.

Consent for publication

Not applicable

Competing interests

The authors declare that they have no competing interests.

References

1. WHO, UNICEF. Low birthweight: Country, Regional and Global Estimates. New

York: Unicef; 2004. 1–31 p.

2. Rosal MC, Wang ML, Moore Simas TA, Bodenlos JS, Crawford SL, Leung K, et al. Predictors of Gestational Weight Gain among White and Latina Women and Associations with Birth Weight. J Pregnancy. 2016;2016:1–11.

3. Said AS, Manji KP. Risk factors and outcomes of fetal macrosomia in a tertiary centre in Tanzania: A case-control study. BMC Pregnancy Childbirth. 2016;16(243):1–8.

4. Law CM. Significance of birth weight for the future. Arch Dis Child Fetal Neonatal Ed. 2002;86:F7–8.

5. Risnes KR, Vatten LJ, Baker JL, Jameson K, Sovio U, Kajantie E, et al. Birthweight and mortality in adulthood: A systematic review and meta-analysis. Int J Epidemiol. 2011;40:647–61.

6. Christian P, Lee SE, Angel MD, Adair LS, Arifeen SE, Ashorn P, et al. Risk of childhood undernutrition related to small-for-gestational age and preterm birth in low- and middle-income countries. Int J Epidemiol. 2013;42(5):1340–55.

7. Gu H, Wang L, Liu L, Luo X, Wang J, Hou F, et al. A gradient relationship between low birth weight and IQ: A meta-analysis. Sci Rep. 2017;7(1):1–13.

8. UNICEF-WHO. UNICEF-WHO Low birthweight estimates: Levels and trends 2000–2015. Geneva; 2019.

9. Parsons TJ, Power C, Logan S, Summerbell CD. Childhood predictors of adult obesity: A systematic review. Int J Obes. 1999;23(8):S1–107.

10. Dabelea D, Pettitt DJ, Hanson RL, Imperatore, Giuseppina Bennett PH, Knowler WC. Birth Weight, Type 2 Diabetes, and Insulin Resistance in Pima Indian Children and Young Adults. Diabetes Care. 1999;22(6):944–950.

11. Magnusson Å, Laivuori H, Loft A, Oldereid NB, Pinborg A, Petzold M, et al. The Association Between High Birth Weight and Long-Term Outcomes—Implications for Assisted Reproductive Technologies: A Systematic Review and Meta-Analysis. Front Pediatr. 2021;9(675775).

12. WHO. WHO recommendations on maternal and newborn care for a positive postnatal experience. World Health Organization. Geneva: World Health Organization; 2022.

13. Zaveri A, Paul P, Saha J, Barman B, Chouhan P. Maternal determinants of low birth weight among Indian children: Evidence from the National Family Health Survey-4, 2015-16. PLoS One. 2020;15(12):1–15.

14. Muthayya S. Maternal nutrition & low birth weight - What is really important? Indian J Med Res. 2009;130(5):600–8.

15. Abubakari A, Kynast-Wolf G, Jahn A. Maternal determinants of birth weight in Northern Ghana. PLoS One. 2015;10(8):1–15.

16. Dubois L, Girard M. Determinants of birthweight inequalities: Population-based study. Pediatr Int. 2006;48(5):470–8.

17. Halileh S, Abu-Rmeileh N, Watt G, Spencer N, Gordon N. Determinants of birthweight; Gender based analysis. Matern Child Health J. 2008;12:606–12.

18. Dubois L, Girard M, Tatone-Tokuda F. Determinants of high birth weight by geographic region in Canada. Chronic Dis Can. 2007;28(1–2):63–70.

19. Voldner N, Frey Frøslie K, Godang K, Bollerslev J, Henriksen T. Determinants of birth weight in boys and girls. Hum Ontog. 2009;3(1):7–12.

20. Nketiah-Amponsah E, Abuosi A, Arthur E. Maternal Socio-economic Status and Childhood Birth weight: A Health Survey in Ghana. Neonatal Care. 2012;

21. Corsi DJ, Neuman M, Finlay JE, Subramanian S V. Demographic and health surveys: A profile. Int J Epidemiol. 2012;41(6):1602–13.

22. Aliaga A, Ren R. Cluster Optimal Sample Size for Demographic and Health Surveys. In: 7th International Conference on Teaching Statistics–ICOTS. 2006. p. 1–6.

23. The Demograhic and Health Survey Program. Dataset Types [Internet]. 2023 [cited 2023 Apr 25]. Available from: https://dhsprogram.com/data/dataset-types.cfm#:~:text=Dataset Types 1 Survey Data In order to,collected in the DHS and AIS surveys.

24. Long JS, Freese J. Regression models for categorical dependent variables using Stata. College Station, Texas: Stata Press; 2006.

25. O’Brien RM. A Caution Regarding Rules of Thumb for Variance A Caution Regarding Rules of Thumb for Variance Inflation Factors. Qual Quant. 2007;41:673–

90.

26. Menard S. Applied Logistic Regression Analysis. 2nd ed. London: Sage Publications; 2001. 111 p.

27. Bayrampour H, Heaman M. Advanced Maternal Age and the Risk of Cesarean Birth : A Systematic Review. Birth. 2010;37(3):219–26.

28. Londero AP, Rossetti E, Pittini C, Cagnacci A, Driul L. Maternal age and the risk of adverse pregnancy outcomes : a retrospective cohort study. BMC Pregnancy Childbirth. 2019;19(1):1–10.

29. Zajacova A, Lawrence EM. The Relationship between Education and Health: Reducing Disparities Through a Contextual Approach. Annu Rev Public Health. 2018;39:273–89.

30. Hahn RA, Truman BI. Education improves public health and promotes health equity. Int J Heal Serv. 2015;45(4):657–78.

31. Raghupathi V, Raghupathi W. The influence of education on health: An empirical assessment of OECD countries for the period 1995-2015. Arch Public Heal. 2020;78(1):1–18.

32. Jewell RT, Triunfo P. The impact of prenatal care on birthweight: the case of Uruguay. Health Econ. 2006;15:1245–50.

33. Manyeh AK, Kukula V, Odonkor G, Ekey RA, Adjei A, Narh-Bana S, et al. Socioeconomic and demographic determinants of birth weight in southern rural Ghana: Evidence from Dodowa Health and Demographic Surveillance System. BMC Pregnancy Childbirth. 2016;16(1):1–9.

34. Zhang F, Shi X, Zhou Y. The impact of health insurance on healthcare utilization by migrant workers in China. Int J Environ Res Public Health. 2020;17(6):1852.

35. Wang W, Temsah G, Mallick L. The impact of health insurance on maternal health care utilization: Evidence from Ghana, Indonesia and Rwanda. Health Policy Plan. 2017;32(3):366–75.

36. Farrell CM, Gottlieb A. The Effect of Health Insurance on Health Care Utilization in the Justice-Involved Population : United States, 2014-2016. Am J Public Health.

2020;110(S1):S78–84.

37. WHO. WHO recommendations on antenatal care for a positive pregnancy experience. Geneva: World Health Organization; 2016.

38. Banerjee S. Determinants of rural-urban differential in healthcare utilization among the elderly population in India. BMC Public Health. 2021;21(1):1–18.

39. Fekadu GA, Ambaw F, Kidanie SA. Fekadu GA, Ambaw F, Kidanie SA. Facility delivery and postnatal care services use among mothers who attended four or more antenatal care visits in Ethiopia: further analysis of the 2016 demographic and health survey. BMC Pregnancy Childbirth. 2019;19(1):64.

40. Chungu C, Makasa M, Chola M, Jacobs CN. Place of Delivery Associated With Postnatal Care Utilization Among Childbearing Women in Zambia. Front Public Heal. 2018;6(94):1–7.

41. Sultana AM. Factors effect on women autonomy and decision-making power within the household in rural communities. J Appl Sci Res. 2011;7(1):18–22.

42. Cannonier C, Burke MG. The Impact of Education on Household Decision-Making Among Women in Sierra Leone. In: The Palgrave Handbook of Africa's Economic Sectors. Cham: Palgrave Macmillan; 2022. p. 217–54.

43. Senarath U, Gunawardena NS. Women's Autonomy in Decision Making for Health Care in South Asia. Asia Pacific J Public Heal. 2009;21(2):137–43.

Appendix

Table 1A: Collinearity diagnostics for the independent variable

Variable	VIF	TI
Child sex	1.00	0.99
Birth type	1.01	0.98
Age	2.33	0.43
Education level	1.55	0.64
Marital Status	1.13	0.88
Residence	1.43	0.70
Parity	2.53	0.40
Health decisions	1.08	0.93
Used ANC	1.27	0.78
Place of delivery	1.51	0.66
Insurance	1.09	0.92
Distance to a health facility	1.12	0.89
Wealth status	1.78	0.56
Currently working	1.05	0.95
Country	1.21	0.82

Note: VIF - variance inflation factor. TI - tolerance indices

Click here to access/download
Supporting Information - Compressed/ZIP File Archive
Data.zip

Factors associated with weighing a child at birth: evidence from 16 sub-Saharan African countries

Alex Bawuah[1], ORCID: 0000-0003-4603-5789

Samuel Ampaw[2], ORCID: 0000-0001-5881-4347

Edward Nketiah-Amponsah[3], ORCID: 0000-0002-9754-1983

[1]Health Economics Research Unit, Aberystwyth Business School, Aberystwyth University, Aberystwyth, Ceredigion, Wales UK, SY23 3DY.

[2]Global Poverty Research Lab, Kellogg School of Management, Northwestern University, Evanston, Illinois, USA.

[*3]Department of Economics, University of Ghana, Legon.

enamponsah@ug.edu.gh

Abstract

Background: Several children from sub-Saharan Africa (SSA) are not weighed at birth. The lack of birthweight data is a significant challenge in monitoring the global prevalence of extreme birthweight, either low or high, and newborn health. This data guides resource allocation and the design of targeted health policies to address neonatal complications and mortalities. This paper explores the demand-side predictors of newborn weighing.

Methods: Data were obtained from the Demographic and Health Surveys (DHS) of 16 countries in SSA, conducted from 2014 to 2021. Multivariate logistic regression was used to achieve the study's objectives.

Results: Approximately 59% of the study population were weighed at birth. This prevalence rate varied widely across the 16 countries, ranging from 23% in Chad to 94% in Gabon. The study documents a positive association between higher socioeconomic ~~and demographic~~ status and the probability of being weighed at birth. Specifically, older women and women with higher education and wealth were more likely to weigh their ~~wards~~ newborns at birth. Also, women who delivered at healthcare facilities and those who used antenatal care had a higher likelihood of weighing their children at birth. Urban residents were more likely to weigh their children at birth. On the contrary, the likelihood of weighing a child at birth decreases with parity ~~(adjusted odds ratio [AOR]=0.94, 95% CI=0.92 - 0.95)~~.

Conclusion: The study highlights the need to target pregnant women of lower socioeconomic ~~and demographic~~ status for interventions aimed at averting severe morbidity and mortality occasioned by conditions of low birthweight.

Keywords: Child; Birthweight; Weighed at birth; sub-Saharan Africa.

Background

Birthweight is the first weight of the foetus or newborn measured after birth [1]. It can be categorized into three: low birthweight (birthweight ≤ 2.5 kg), normal birthweight (birthweight between 2.5kg – 4.0kg), and macrosomia or high birthweight (birthweight ≥> 4.0 kg) [2,3]. A child's birthweight is an essential indicator of their health and development, as it can affect their short- and long-term outcomes [4]. For instance, low birthweight babies are at a higher risk of developing chronic diseases [5], stunted growth [6], lower IQ [7], and reduced life expectancy [8]. High birthweight is associated with high risks for certain malignancies in childhood, hypertension in childhood, psychiatric disorders, type 1 and 2 diabetes, breast cancer, and obesity [9–11].

Given the risk factors associated with high and low birthweight, the WHO recommends that babies be weighed during postnatal care [12]. Weighing children at birth has several merits. First, knowledge about the birthweight provides vital information about a child's health [5]. It guides medical care and interventions such as medications and feeding plans , such as medications and feeding plans, to promote the best possible outcomes for the child's health and development. For instance, babies with low or high birthweight may need to be fed differently or be given particular medications compared to babies with normal birthweight. Second, weighing newborns helps monitor the prevalence of low or high birthweight [8]. Third, a child's birthweight can give important information about the mother's health. Studies have shown that mothers who have poor nutrition, anaemic conditionconditions, and are underweight are more likely to have low birthweight babies [13,14]. Fourth, studies relating to a child's birthweight have often been centred around the factors that influence a child's birthweight [15–20]. Researchers were able to conduct these studies because the children were weighed at birth, and their birthweights were recorded. These studies could not have been conducted undertaken if children were not weighed at birth.

The challenge, however, is that many newborns are not weighed at birth. For instance, in 2015, nearly one-third of newborns (39.7 million) globally were not weighed at birth, with Africa recording the highest percentage of newborns without a recorded birthweight (51.7%, or 21.5 million) [8]. This situation places a significant challenge on monitoring the prevalence of low or high birthweight and the health of newborns due to the lack of birthweight data for many children. This study, therefore, aims to investigate the factors associated with weighing a child at birth in sub-Saharan Africa (SSA).

Various factors, including individual sociodemographic, maternal, household, and community-level factors, can influence the decision to weigh a child at birth. Understanding these factors can guide policymakers ~~to design or implement~~ implement policies that will encourage the weighing of newborns. To the best of our knowledge, there has been no published literature on this topic. This study is the first to explore the factors influencing the decision to weigh newborns.

Methods

Data source

The study used data from the Demographic and Health Surveys (DHS) of 16 countries in SSA conducted from 2014 to 2021 (these are countries with high neonatal mortality rates ~~and~~ for which data were available ~~data~~). The DHS is a nationwide survey conducted in over 85 low- and middle-income countries worldwide and follows a consistent protocol and terminology across all countries [21]. It employs a structured questionnaire to gather information on various health indicators, including maternal and child health, fertility, family planning utilization, morbidity, and mortality [21]. The DHS uses a two-stage sampling technique to collect data, starting with selecting enumeration areas based on each country's sampling frame, followed by the selection~~g~~ of households from each enumeration area. Detailed information on the sampling and data collection methods can be found in the work of Aliaga and Ren [22].

This research employed the children's dataset (KR file) from the DHS surveys. The KR file contains a single entry for each child born to the interviewed women within the five years before the survey [23]. The total sample size of children (aged 0 to 5 years) from the sixteen countries in the specified duration was 183,541.

Variables

The study's outcome variable is whether the child was weighed at birth. In the survey, the respondents were asked if the child was weighed at birth. The response was coded as "1 = Yes" and "0 = No" for children who were weighed and those who were not weighed at birth, respectively.

The study used a total of 13 explanatory variables. The variables include the respondent's age, woman's highest level of education, marital status, residence, parity, decisions about woman's health, used antenatal care (ANC), place of delivery, covered by insurance, household wealth status, birth type, sex of the child, currently working status and distance to a healthcare facility.

Respondent's age was coded in a five-interval scale beginning from 15 to 49. as "15–19", "20–24", "25–29", "30–34", "35–39", "40–44", and "45–49". Women's education was coded as "none", "primary", "secondary", and "higher". Marital status was coded as "married" and "otherwise". The residence was coded as "rural" and "urban". Parity indicates the number of times the woman has had a live birth. Decisions about women's health were coded as "woman alone", "together with partner/husband", and "husband/partner alone". ANC use was coded as "yes" and "no". The place of delivery (childbirth) was coded as "health facility" and "otherwise". Covered by insurance was coded as "yes" and "no". Household wealth status was coded as "poorest", "poorer", "middle", "richer", and "richest". The birth type was coded as "single birth" and "multiple births". Child sex was coded as "male" and "female". Distance to healthcare facilities was coded as "a big problem" and "not a big problem" (in the survey, the women were asked whether distance to the healthcare facility was a big problem or not a big problem).

The year variable (the year in which the survey was conducted) was initially included in the model to account for potential temporal effects. However, we found that it was perfectly collinear with the country variable. Given this collinearity, the year variable was omitted from the final model. Furthermore, birth order was not included in the modelthe model did not include birth order due to collinearity with the parity variable.

Data analysis

The data were analysed using STATA version 16. Frequency tables were used to describe the data (Table 2). Given the binary nature of the outcome dependent variable, the study applied logistic regressions to evaluate the determinants of measuring a child's weight at birth [24]. At first, a bivariate logistic regression analysis was conducted to estimate the unadjusted odds ratios (ORs) for each determinant. Afterwards, a multivariable logistic regression was performed to estimate the adjusted odds ratios (AORs) for all determinants, taking into accountconsidering clustering (at the country level), and sampling weights. Missing observations were not included in the regression analysis. We handled missing data using listwise deletion;, thus, observations with missing values in any of the variables included in the regression model were excluded from the analysis. This approach ensures consistency across all model estimates.

Formatted: Font: 12 pt

Formatted: Font: 12 pt

Prior to the regression analysis, we ~~did~~ conducted a diagnostic test for collinearity between the independent variables by looking at the variance inflation factor (VIF) and tolerance indices (TI) of the independent variables. VIF and TI are measures used to assess the level of multi-collinearity between the *i*th independent variable and other independent variables in a regression model [25]. VIFs above 5 and TIs less than 0.20 are considered signs of multi-collinearity [26] . The VIFs for all the independent variables used in the study were below 5. Similarly, the TIs were above 0.20. These results are provided in Table A1 in the appendix.

Ethical Consideration

The data used for this study, Demographic and Health Survey (DHS) is secondary, and its usage does not require ethical clearance. Measure DHS, which is responsible for undertaking standardized Demographic and Health Surveys in over 90 countries ~~duly obtained ethical clearance from the requisite institutions from all the 16 countries included in this study~~, duly obtained ethical clearance from the requisite institutions in all 16 countries included in this study, as well as the Institutional Review Board of the ICF International. In this regard, ethical approval and ~~consents of participants to participate in the study is~~consent of participants to participate in the study are not applicable. It is worth noting that all data are anonymous and confidential, so there is no way of identifying individuals in the database.

Results

Descriptive statistics

As shown in Table 1, the sample size for the study was 183,541. Almost 60% of the children were weighed at birth. Rwanda had the highest percentage of children weighed at birth (95.12%), whereas Ethiopia had the lowest (27.48%). Most of the countries recorded a less than 70% prevalence of babies weighed at birth (Angola – 55.14%; Cameroon – 69.19%; Ethiopia – 27.48%; Gambia 63.81%; Guinea – 51.06%; Kenya – 59.51%; Liberia – 65.83%; Madagascar – 38.53%; Malawi – 52.66%; Nigeria – 29.28%).

Table 1 – Sample size characteristics

Country	Year of survey	Number of children	% of Children weighed at birth
Angola	2015-2016	14322	55.14
Benin	2017-2018	13589	76.48

Cameroon	2018	9733	69.19
Ethiopia	2016	10641	27.48
Gambia	2019-2020	5447	63.81
Ghana	2014	8362	86.04
Guinea	2018	7951	51.06
Kenya	2014	9967	59.51
Liberia	2019-2020	5704	65.83
Madagascar	2021	12499	38.53
Malawi	2015-2016	9940	52.66
Mali	2018	17286	91.7
Nigeria	2018	33924	29.28
Rwanda	2019-2020	8092	95.12
Senegal	2019	6125	76.91
Zambia	2018	9959	83.2
Total		183541	59.33

Table 2 presents the results of the descriptive summary of the sample. It shows that most of the children are males (50.73%). Also, a higher proportion of the respondents are between 25 and 29 years old (26.79%). Likewise, most of the respondents were married (74.62%), rural residents (67.99%), had single birth (96.47%), used ANC (87.16), and reported that distance to a healthcare facility was not a big problem (61.02a single birth (96.47%), used ANC (87.16%), and reported that distance to a healthcare facility was not a big problem (61.02%). The majority ofMost of the respondents did not have insurance (91.5%).

Furthermore, most of the children who were weighed at birth were delivered in a healthcare facility (91.71%), whereas those who were not weighed were delivered elsewhere (83.18%), mostly at home (81.74%). Similarly, a higher proportion of those who weighed their children had primary education (36.61%), whereas those who did not weigh their children had no education (59.18%). Likewise, a higher proportion of those who weighed their children belonged to the wealthiest wealth category (21.6%), whereas those who did not weigh their children belonged to the poorest category (39.95%). Also, the mean parity of those who did not weigh their children is higher (4.45) than that of those who weighed their children (3.53).

Table 2 – Descriptive statistics of the sample

Variable	Pooled Sample	Weighed	Not Weighed	
	Frequency (%)	Frequency (%)	Frequency (%)	χ^2/ Diff.t-test
**Child's Characteristics**				
Weighed at birth (*n* =183541)				
Yes	108892 (59.33)			
No	74649 (40.67)			
Missing = 0 (0.00%)				
Child's sex (*n* =183541)				
Male	93104 (50.73)	55527 (50.99)	37577 (50.34)	0.006
Female	90437 (49.27)	53365 (49.01)	37072 (49.66)	
Missing = 0 (0.00%)				
Birth type (*n* =183541)				
Single birth	177062 (96.47)	104721 (96.17)	72350 (96.92)	<0.001
Multiple births	6473 (3.53)	4173 (3.83)	2301 (3.08)	
Missing = 0 (0.00%)				
**Mother's Characteristics**				
Age (*n* =183541)				
15 – 19	11510 (6.27)	6733 (6.18)	4777 (6.40)	<0.001
20 – 24	41277 (22.49)	24796 (22.77)	16481 (22.08)	
25 – 29	49173 (26.79)	29188 (26.80)	19985 (26.77)	
30 – 34	38206 (20.82)	23069 (21.19)	15137 (20.28)	
35 – 39	27072 (14.75)	16233 (14.91)	10839 (14.52)	
40 – 44	12267 (6.68)	6885 (6.32)	5382 (7.21)	
45 – 49	4036 (2.20)	1988 (1.83)	2048 (2.74)	
Missing = 0 (0.00%)				
Level of education (*n* =183539)				
None	73968 (40.30)	29790 (27.36)	44178 (59.18)	<0.001
Primary	59385 (32.36)	39865 (36.61)	19520 (26.15)	
Secondary	43334 (23.61)	33059 (30.36)	10277 (13.77)	
Higher	6853 (3.73)	6179 (5.68)	674 (0.90)	
Missing = 2 (0.00%)				
Marital Status (*n* =183541)				
Married	136961 (74.62)	78307 (71.91)	58654 (78.57)	<0.001
Otherwise	46580 (25.38)	30585 (28.09)	15995 (21.43)	
Missing = 0 (0.00%)				
Residence (*n* =183541)				
Urban	58753 (32.01)	45620 (41.89)	13123 (17.58)	<0.001
Rural	124798 (67.99)	63272 (58.11)	61526 (82.42)	
Missing = 0 (0.00%)				
Parity (*n* =183541)	*Mean* = 3.91	*Mean* = 3.53	*Mean* = 4.45	<0.001
Missing = 0 (0.00%)				
Health decisions (*n* =159575)				
Alone	26163 (16.40)	17312 (18.67)	8851 (13.24)	<0.001
Together with partner/husband	63640 (39.88)	39815 (42.94)	23825 (35.64)	
Husband/partner alone	69772 (43.72)	35600 (38.39)	34172 (51.12)	
Missing = 23966 (13.06%)				
Used ANC (*n* =125712)				
Yes	109568 (87.16)	77486 (97.95)	32082 (68.84)	<0.001
No	16144 (12.84)	1625 (2.05)	14519 (31.16)	
Missing = 57829 (31.51%)				
Place of delivery (*n* =183530)				
Health facility	112413 (61.25)	99857 (91.71)	12556 (16.82)	<0.001
Otherwise	71117 (38.75)	9028 (8.29)	62089 (83.18)	
Missing = 11 (0.01%)				
Insurance (*n* =177413)				
Yes	15077 (8.50)	12689 (12.18)	2388 (3.26)	<0.001
No	162336 (91.50)	91489 (87.82)	70847 (96.74)	
Missing = 6128 (3.34%)				

Distance to a facility (*n* =183535)				
A big problem	71538 (38.98)	35623 (32.72)	35915 (48.11)	<0.001
Not a big problem	111997 (61.02)	73265 (67.28)	38732 (51.89)	
Missing = 6 (0.00%)				
Currently working (*n* =183,525)				
Yes	117785 (64.18)	71339 (65.52)	46446 (62.22)	
No	65740 (35.82)	37540 (34.48)	28200 (37.78)	<0.001
Currently working (*n* =183,525)				
**Household Characteristics**				
Wealth status (*n* =183541)				
Poorest	48380 (26.36)	18561 (17.05)	29819 (39.95)	<0.001
Poorer	41210 (22.45)	20871 (19.17)	20339 (27.25)	
Middle	36739 (20.02)	22903 (21.03)	13836 (18.53)	
Wealthier	30732 (16.74)	23032 (21.15)	7700 (10.31)	
Wealthiest	26480 (14.43)	23525 (21.60)	2955 (3.96)	
Missing = 0 (0.00%)				

Determinants of weighing children at birth

Table 3 presents the findings from both the bivariate and multivariable logistic regression analyses. Results of the multivariable logistic regression show that age, parity, place of delivery, residence, education, wealth status, the person(s) who decides on the woman's healthcare, ANC utilisation, distance to a health facility, and having insurance were significant determinants of weighing children at birth. The odds of weighing children at birth for respondents aged between 20 – 24, 25 – 29, 30 – 34, 35 – 39, 40 – 44, and 45 – 49 were respectively 1.14 (95% CI=1.01 - 1.29), 1.30 (95% CI=1.03 - 1.63), 1.57 (95% CI=1.17 - 2.09), 1.85 (95% CI=1.35 - 2.54), 1.91 (95% CI=1.32 - 2.76), and 1.67 (95% CI=1.14 - 2.44) times higher than those between the ages of 15 – 19. Furthermore, relative to the uneducated, the odds of weighing children at birth were 1.38 (95% CI=1.19 - 1.60), 1.64 (95% CI=1.41 - 1.91), and 2.68 (95% CI=2.33 - 3.08) times higher for women with primary, secondary, and higher education, respectively. Similarly, the probability of weighing children at birth was 1.20 (95% CI=1.07 - 1.35), 1.48 (95% CI=1.22 - 1.78), 2.05 (95% CI=1.56 - 2.70), and 3.91 (95% CI=2.96 - 5.17~~) times~~) times higher for those from poorer, middle, wealthier and wealthiest households, respectively, compared to those from the poorest households. ~~Those~~ Women who delivered at healthcare facilities were~~are~~ more likely to weigh their children at birth (AOR=52.29, 95% CI=38.43 - 71.16). Similarly, women who used ANC during pregnancy ~~are~~ were more likely to weigh their children at birth (AOR=4.62, 95% CI=3.76 - 5.67). Urban residents were more likely to weigh their children at birth (AOR=2.29, 95% CI=1.79 - 2.91) than rural residents. Also, the insured are more likely to weigh their children at birth (AOR=1.32, 95% CI=1.09 - 1.61). In addition, women who make decisions about their healthcare alone and those who decide jointly with their husbands/partners are 1.28 (95% CI=1.04 - 1.57) and 1.22 (95% CI=1.01 - 1.46) times

more likely than women whose husbands/partners alone make decisions about their healthcare. Furthermore, those who reported that distance to the healthcare facility is not a big problem ~~are more likely to weigh their children (AOR=1.09, 95% CI=1.00 - 1.19)~~ ~~than their counterparts that had difficulty in accessing~~ were more likely to weigh their children (AOR=1.09, 95% CI=1.00 - 1.19) than their counterparts who had difficulty accessing the healthcare facility. Moreover, the likelihood of weighing children at birth decreases as parity increases (AOR=0.94, 95% CI=0.90 - 0.98). Furthermore, children from other countries are less likely to be weighed compared to children from Rwanda.

The interactions between residence and place of birth showed that urban residents who give birth in healthcare facilities are 54.84 times more likely to weigh their children compared to rural residents who give birth at other places. Similarly, women who used ANC services and gave birth in a healthcare facility ~~are~~ were 166.69 times more likely to weigh their children than those who did not use ANC and gave birth elsewhere. Also, compared to rural residents with no education, urban residents with secondary levels of education and urban residents with higher levels of education are 5.12 and 8.80 (respectively) times more likely to weigh their children.

Table 3 – Determinants of weighing children at birth in sub-Saharan Africa

Variables	Unadjusted OR	Adjusted OR
**Child's Characteristics**		
Child sex (Ref: Male)		
Female	0.97 (0.96 - 1.00)**	1.02 (0.97 - 1.07)
Birth type (Ref: Single)		
Multiple	1.21 (1.03 - 1.41)**	1.03 (0.89 - 1.20)
**Mother's Characteristics**		
Age at birth (Ref: 15 – 19)		
20 – 24	1.10 (0.95 - 1.28)	1.14 (1.01 - 1.29)**
25 – 29	1.08 (0.83 - 1.40)	1.30 (1.03 - 1.63)**
30 – 34	1.13 (0.84 - 1.50)	1.57 (1.17 - 2.09)***
35 – 39	1.11 (0.80 - 1.55)	1.85 (1.35 - 2.54)***
40 – 44	0.94 (0.69 - 1.27)	1.91 (1.32 - 2.76)***
45 – 49	0.68 (0.52 - 0.90)***	1.67 (1.14 - 2.44)***
Education level (Ref: None)		
Primary	3.11 (1.69 - 5.72)***	1.38 (1.19 - 1.60)***
Secondary	5.19 (3.33 - 8.07)***	1.64 (1.41 - 1.91)***
Higher	15.16 (8.70 - 26.41)***	2.68 (2.33 - 3.08)***
Marital Status (Ref: Otherwise)		
Married	0.64 (0.36 - 1.13)	1.15 (0.84 - 1.57)
Residence (Ref: Rural)		
Urban	3.78 (2.21 - 6.47)***	2.29 (1.79 - 2.91)***
Parity	0.84 (0.81 - 0.87)***	0.94 (0.90 - 0.98)***
Health decisions (Ref: Husband/partner		

alone)		
Woman Alone	1.97 (1.02 - 3.82)**	1.28 (1.04 - 1.57) **
Together with partner/husband	1.64 (0.81 - 3.32)	1.22 (1.01 - 1.46) **
Used ANC (Ref: No)		
Yes	21.76 (16.25 - 29.15)***	4.61 (3.76 - 5.67)***
Place of delivery (Ref: Otherwise)		
Health facility	54.60 (34.47 - 86.48)***	52.29 (38.43 - 71.16)***
Insurance (Ref: No)		
Yes	4.35 (1.48 - 12.85)***	1.32 (1.09 - 1.61)***
Distance to health facility (Ref: big problem)		
Not a big problem	1.79 (1.14 - 2.83)**	1.09 (1.00 - 1.19)*
Currently working (Ref: No)		
Yes	1.19 (0.77 - 1.83)	1.01 (0.94 - 1.09)
**Household Characteristics**		
Wealth status (Ref: Poorest)		
Poorer	1.51 (1.24 - 1.84)***	1.20 (1.07 - 1.35)***
Middle	2.40 (1.67 - 3.43)***	1.48 (1.22 - 1.78)***
Wealthier	4.52 (2.85 - 7.16)***	2.05 (1.56 - 2.70)***
Wealthiest	12.77 (7.89 - 20.67)***	3.91 (2.96 - 5.17)***
**Countries** (Ref: Rwanda)		
Angola	0.08 (0.08 - 0.08)***	0.43 (0.35 - 0.53)***
Benin	0.17 (0.17 - 0.17)***	0.21 (0.16 - 0.28)***
Cameroon	0.11 (0.11 - 0.11)***	0.23 (0.19 - 0.27)***
Ethiopia	0.01 (0.01 - 0.01)***	0.05 (0.04 - 0.06)***
Ghana	0.10 (0.10 - 0.10)***	0.10 (0.09 - 0.11)***
Gambia	0.49 (0.49 - 0.49)***	0.87 (0.70 - 1.09)
Guinea	0.06 (0.06 - 0.06)***	0.19 (0.15 - 0.24)***
Kenya	0.10 (0.10 - 0.10)***	0.22 (0.19 - 0.26)***
Liberia	0.11 (0.11 - 0.11)***	0.08 (0.06 - 0.10)***
Madagascar	0.03 (0.03 - 0.03)***	0.09 (0.08 - 0.11)***
Mali	0.06 (0.06 - 0.06)***	0.10 (0.07 - 0.14)***
Malawi	0.53 (0.53 - 0.53)***	0.57 (0.46 - 0.71)***
Nigeria	0.02 (0.02 - 0.02)***	0.03 (0.02 - 0.04)***
Senegal	0.21 (0.21 - 0.21)***	0.35 (0.27 - 0.46)***
Zambia	0.29 (0.29 - 0.29)***	0.35 (0.28 - 0.44)***
**Interactions**		
Residence & Birthplace (Ref: Rural & Otherwise)		
Urban*Health facility		0.46 (0.36 - 0.59)***
ANC & Birthplace (Ref: No ANC & Otherwise)		
Used ANC* Health facility		0.69 (0.54 - 0.87)***
Residence & Education ((Ref: Rural & No education)		
Urban*Primary		1.13 (0.93 - 1.38)
Urban*Secondary		1.37 (1.11 - 1.70)***
Urban*Higher		1.44 (1.07 - 1.94)**
Constant		0.13 (0.10 - 0.17)***

Log pseudolikelihood	-2.821e+10
Pseudo R^2	0.59
Observations	102,709

Note: OR = odds ratio; Ref = Reference group. Sampling weights and clustering (at country level) accounted for in the regression analysis. 95% confidence interval (CI) in parentheses. *** p<0.01, ** p<0.05, * p<0.10

Discussion

The study examined the factors associated with weighing a child at birth using the most recent DHS data from 16 SSA countries. We found that older women were more likely to weigh their children than younger women. Studies have found that older women have a higher chance of encountering complications during pregnancy, which could have ~~a negative~~an adverse effect on their children [25,26]. ~~. Knowing this information, older women may likely want to weigh their children to safeguard their children's~~ Knowing this information, older women may want to weigh their children to safeguard their health.

Educated women were more likely to weigh their children than uneducated women. This finding may be because the educated are better informed about the benefits of weighing children at birth than the uneducated. For instance, women with higher levels of education may learn about the importance of weighing children at birth. Moreover, studies have documented the correlation between education and health outcomes for women and children [27–29].

Parity was negatively correlated with the odds of weighing a child. The study revealed that the likelihood of weighing a child at birth decreases as parity increases. This finding may be linked with the observation that women with more pregnancy and birthing experience can often produce healthy babies [30,31]. Therefore, women with more parity may not see the need to weigh their children.

The results from the study further revealed that women covered by health insurance are more likely to weigh their children at birth. Health insurance may cover the cost of healthcare services, which ~~includes~~ include weighing a child, hence encouraging women, especially those with low incomes, to weigh their children. Moreover, insurance increases access to healthcare services [32–34]; hence, women ~~who have~~with insurance may be more likely to use healthcare services such as weighing their children at birth.

The study further revealed a positive association between wealth status and weighing children at birth. The likelihood of weighing a child at birth increases with household wealth. One

possible explanation behind this finding could be the cost of transportation to healthcare facilities. The poor may be unable to afford the ~~transportation cost of transporting their babies to the~~ cost of transporting their babies to healthcare facilities to have their weight checked, unlike the wealthy, who can afford it. Another possible explanation may be that low-income families prioritize meeting their fundamental daily necessities, resulting in fewer resources for healthcare (such as weighing their babies) compared to affluent households. This finding (the positive correlation between wealth and weighing of children at birth) suggests implementing anti-poverty measures to encourage weighing children at birth.

The use of ANC was also positively correlated with the weighing of children at birth. This finding may be linked to the components of ANC. ANC includes health education and promotion, prevention and management of pregnancy-related or concurrent diseases, and risk detection [35]. Thus, women who use ANC are educated or informed about the importance of weighing children at birth, perhaps explaining why they have a higher chance of weighing their children.

Place of residence also emerged as a significant determinant of weighing children at birth. The study found that rural residents are less likely to weigh their children. Individuals who live in urban areas have better access to a range of healthcare facilities and healthcare personnel, which makes it easier for them to access healthcare services [36], including the weighing of their children.

Women who gave birth at healthcare facilities were more likely to weigh their children at birth. There are two possible explanations for this finding. The first relates to the availability of resources at the healthcare facilities, such as the tools/equipment used to weigh children and the personnel who can weigh the children. The second could be linked to the fact that women who deliver at healthcare facilities are more likely to receive postnatal care, which also includes weighing children [37,38].

One of the strengths of this study is that it is the first to investigate the factors that influence the decision to weigh a child at birth. This is a ~~major~~ significant contribution to the literature, given that there has been no published literature on this topic. Another strength of this study is that it used nationally representative data from 16 sub-Saharan African countries to examine the factors influencing the decision to weigh a child at birth. Therefore, lessons from this study can be relevant to other countries in sub-Saharan Africa (due to the similarity in the healthcare systems and population characteristics among sub-Saharan African countries).

One ~~of the limitations~~limitation of this study is that potential supply-side predictors such as the healthcare facility, personnel, and weighing equipment availability were not accounted for due to data limitations. Future studies can explore the extent to which these supply-side variables influence the decision to weigh a newborn. Another limitation is that the study only establishes association, not causality. Nonetheless, the study offers a foundation for more rigorous analysis in future studies.

Conclusion

The study investigated the factors associated with weighing a child at birth using nationally representative data from 16 SSA countries. The overall prevalence of weighing children at birth was 59%. This ranged from 27.48% in Ethiopia to 95.12% in Rwanda. Results from the study's multivariable regression showed that old age, having an education, being married, giving birth in a healthcare facility, having insurance, using ANC, living in urban areas, having autonomy in making healthcare decisions and having a higher wealth status were positively correlated (significantly) with weighing children at birth. However, higher parity was negatively correlated (significantly) with weighing children at birth.

The study's findings have some important policy implications. The finding that health facility delivery and ~~the use of~~using ANC ~~are positively correlated~~positively correlate with weighing a child at birth suggests ~~the need to implement~~implementing policies/programs to encourage ANC use and facility delivery. One such policy could be creating awareness programs to educate women of childbearing age about the importance of using ANC and facility delivery. This can be done through local media, social media, and other media platforms. Furthermore, the study also provides support for women empowerment programmes. The statistical significance of having autonomy in healthcare decisions and formal education suggests the need to institute policies that promote girl child education, such as providing scholarships for brilliant but needy girls. Moreover, the literature highlights the positive correlation between education and the autonomy of women in decision-making [39–41]. In ~~the adoption of~~adopting these interventions, special considerations could be given to the poor and rural residents.

List of abbreviations

OR: Odds Ratio

AOR: Adjusted Odds Ratio

CI: Confidence Interval
ANC: Antenatal Care
SSA: Sub-Sahara Africa
DHS: Demographic and Health Survey
WHO: World Health Organisation
IQ: Intelligence Quotient
VIF: Variance Inflation Factor
TI: Tolerance Indices

Declarations

Acknowledgement

We thank the Demographic and Health Survey (DHS) program for granting us access to the DHS data for the 16 countries.

Authors' Contributions

AB: Conceptualisation, Methodology, Validation, Formal Analysis, Investigation, Resources, Data Curation, Writing—Original Draft, Writing---Review & Editing, Visualisation, Project Administration.

SA: Methodology, Validation, Formal Analysis, Investigation, Resources, Data Curation, Writing---Review & Editing, Visualisation.

ENA: Conceptualisation, Methodology, Formal Analysis, Validation, Resources, Writing---Review & Editing, Visualisation.

Funding

No funding was received for conducting this study.

Availability of Data

The data is owned by a third party – Demographic and Health Survey (DHS). The data are publicly available on the DHS website (www.dhsprogram.com). Interested parties can request for the data from DHS.

Consent for publication

Not applicable

Competing interests

The authors declare that they have no competing interests.

References

1. WHO, UNICEF. Low birthweight: Country, Regional and Global Estimates. New York: Unicef; 2004. 1–31 p.

2. Rosal MC, Wang ML, Moore Simas TA, Bodenlos JS, Crawford SL, Leung K, et al. Predictors of Gestational Weight Gain among White and Latina Women and Associations with Birth Weight. J Pregnancy. 2016;2016:1–11.

3. Said AS, Manji KP. Risk factors and outcomes of fetal macrosomia in a tertiary centre in Tanzania: A case-control study. BMC Pregnancy Childbirth. 2016;16(243):1–8.

4. Law CM. Significance of birth weight for the future. Arch Dis Child Fetal Neonatal Ed. 2002;86:F7–8.

5. Risnes KR, Vatten LJ, Baker JL, Jameson K, Sovio U, Kajantie E, et al. Birthweight and mortality in adulthood: A systematic review and meta-analysis. Int J Epidemiol. 2011;40:647–61.

6. Christian P, Lee SE, Angel MD, Adair LS, Arifeen SE, Ashorn P, et al. Risk of childhood undernutrition related to small-for-gestational age and preterm birth in low- and middle-income countries. Int J Epidemiol. 2013;42(5):1340–55.

7. Gu H, Wang L, Liu L, Luo X, Wang J, Hou F, et al. A gradient relationship between low birth weight and IQ: A meta-analysis. Sci Rep. 2017;7(1):1–13.

8. UNICEF-WHO. UNICEF-WHO Low birthweight estimates: Levels and trends 2000–2015. Geneva; 2019.

9. Parsons TJ, Power C, Logan S, Summerbell CD. Childhood predictors of adult obesity: A systematic review. Int J Obes. 1999;23(8):S1–107.

10. Dabelea D, Pettitt DJ, Hanson RL, Imperatore, Giuseppina Bennett PH, Knowler WC. Birth Weight, Type 2 Diabetes, and Insulin Resistance in Pima Indian Children and Young Adults. Diabetes Care. 1999;22(6):944–950.

11. Magnusson Å, Laivuori H, Loft A, Oldereid NB, Pinborg A, Petzold M, et al. The Association Between High Birth Weight and Long-Term Outcomes—Implications for Assisted Reproductive Technologies: A Systematic Review and Meta-Analysis. Front Pediatr. 2021;9(675775).

12. WHO. WHO recommendations on maternal and newborn care for a positive postnatal experience. World Health Organization. Geneva: World Health Organization; 2022.

13. Zaveri A, Paul P, Saha J, Barman B, Chouhan P. Maternal determinants of low birth weight among Indian children: Evidence from the National Family Health Survey-4, 2015-16. PLoS One. 2020;15(12):1–15.

14. Muthayya S. Maternal nutrition & low birth weight - What is really important? Indian J Med Res. 2009;130(5):600–8.

15. Abubakari A, Kynast-Wolf G, Jahn A. Maternal determinants of birth weight in Northern Ghana. PLoS One. 2015;10(8):1–15.

16. Dubois L, Girard M. Determinants of birthweight inequalities: Population-based study. Pediatr Int. 2006;48(5):470–8.

17. Halileh S, Abu-Rmeileh N, Watt G, Spencer N, Gordon N. Determinants of birthweight; Gender based analysis. Matern Child Health J. 2008;12:606–12.

18. Dubois L, Girard M, Tatone-Tokuda F. Determinants of high birth weight by geographic region in Canada. Chronic Dis Can. 2007;28(1–2):63–70.

19. Voldner N, Frey Frøslie K, Godang K, Bollerslev J, Henriksen T. Determinants of birth weight in boys and girls. Hum Ontog. 2009;3(1):7–12.

20. Nketiah-Amponsah E, Abuosi A, Arthur E. Maternal Socio-economic Status and Childhood Birth weight: A Health Survey in Ghana. Neonatal Care. 2012;

21. Corsi DJ, Neuman M, Finlay JE, Subramanian S V. Demographic and health surveys:

A profile. Int J Epidemiol. 2012;41(6):1602–13.

22. Aliaga A, Ren R. Cluster Optimal Sample Size for Demographic and Health Surveys. In: 7th International Conference on Teaching Statistics–ICOTS. 2006. p. 1–6.

23. The Demograhic and Health Survey Program. Dataset Types [Internet]. 2023 [cited 2023 Apr 25]. Available from: https://dhsprogram.com/data/dataset-types.cfm#:~:text=Dataset Types 1 Survey Data In order to,collected in the DHS and AIS surveys.

24. Long JS, Freese J. Regression models for categorical dependent variables using Stata. College Station, Texas: Stata Press; 2006.

25. O'Brien RM. A Caution Regarding Rules of Thumb for Variance A Caution Regarding Rules of Thumb for Variance Inflation Factors. Qual Quant. 2007;41:673–90.

26. Menard S. Applied Logistic Regression Analysis. 2nd ed. London: Sage Publications; 2001. 111 p.

27. Bayrampour H, Heaman M. Advanced Maternal Age and the Risk of Cesarean Birth : A Systematic Review. Birth. 2010;37(3):219–26.

28. Londero AP, Rossetti E, Pittini C, Cagnacci A, Driul L. Maternal age and the risk of adverse pregnancy outcomes : a retrospective cohort study. BMC Pregnancy Childbirth. 2019;19(1):1–10.

29. Zajacova A, Lawrence EM. The Relationship between Education and Health: Reducing Disparities Through a Contextual Approach. Annu Rev Public Health. 2018;39:273–89.

30. Hahn RA, Truman BI. Education improves public health and promotes health equity. Int J Heal Serv. 2015;45(4):657–78.

31. Raghupathi V, Raghupathi W. The influence of education on health: An empirical assessment of OECD countries for the period 1995-2015. Arch Public Heal. 2020;78(1):1–18.

32. Jewell RT, Triunfo P. The impact of prenatal care on birthweight: the case of Uruguay. Health Econ. 2006;15:1245–50.

33. Manyeh AK, Kukula V, Odonkor G, Ekey RA, Adjei A, Narh-Bana S, et al. Socioeconomic and demographic determinants of birth weight in southern rural Ghana: Evidence from Dodowa Health and Demographic Surveillance System. BMC Pregnancy Childbirth. 2016;16(1):1–9.

34. Zhang F, Shi X, Zhou Y. The impact of health insurance on healthcare utilization by migrant workers in China. Int J Environ Res Public Health. 2020;17(6):1852.

35. Wang W, Temsah G, Mallick L. The impact of health insurance on maternal health care utilization: Evidence from Ghana, Indonesia and Rwanda. Health Policy Plan. 2017;32(3):366–75.

36. Farrell CM, Gottlieb A. The Effect of Health Insurance on Health Care Utilization in the Justice-Involved Population : United States, 2014-2016. Am J Public Health. 2020;110(S1):S78–84.

37. WHO. WHO recommendations on antenatal care for a positive pregnancy experience. Geneva: World Health Organization; 2016.

38. Banerjee S. Determinants of rural-urban differential in healthcare utilization among the elderly population in India. BMC Public Health. 2021;21(1):1–18.

39. Fekadu GA, Ambaw F, Kidanie SA. Fekadu GA, Ambaw F, Kidanie SA. Facility delivery and postnatal care services use among mothers who attended four or more antenatal care visits in Ethiopia: further analysis of the 2016 demographic and health survey. BMC Pregnancy Childbirth. 2019;19(1):64.

40. Chungu C, Makasa M, Chola M, Jacobs CN. Place of Delivery Associated With Postnatal Care Utilization Among Childbearing Women in Zambia. Front Public Heal. 2018;6(94):1–7.

41. Sultana AM. Factors effect on women autonomy and decision-making power within the household in rural communities. J Appl Sci Res. 2011;7(1):18–22.

42. Cannonier C, Burke MG. The Impact of Education on Household Decision-Making Among Women in Sierra Leone. In: The Palgrave Handbook of Africa's Economic Sectors. Cham: Palgrave Macmillan; 2022. p. 217–54.

43. Senarath U, Gunawardena NS. Women's Autonomy in Decision Making for Health Care in South Asia. Asia Pacific J Public Heal. 2009;21(2):137–43.

Appendix

Table 1A: Collinearity diagnostics for the independent variable

Variable	VIF	TI
Child sex	1.00	0.99
Birth type	1.01	0.98
Age	2.33	0.43
Education level	1.55	0.64
Marital Status	1.13	0.88
Residence	1.43	0.70
Parity	2.53	0.40
Health decisions	1.08	0.93
Used ANC	1.27	0.78
Place of delivery	1.51	0.66
Insurance	1.09	0.92
Distance to a health facility	1.12	0.89
Wealth status	1.78	0.56
Currently working	1.05	0.95
Country	1.21	0.82

Note: VIF - variance inflation factor. TI - tolerance indices
