# Supplementary material for: Factors associated with weighing a child at birth: Evidence from 16 sub-Saharan African countries
Source: PLoS One. 2025 Jul 22;20(7):e0328463. doi: 10.1371/journal.pone.0328463 (PMC12282927; doi:10.1371/journal.pone.0328463)
Supplement: Attachment — Submitted filename: Response to Reviewers.docx [file pone.0328463.s005.docx]

**Dear Editor,**

Thank you sincerely for the positive feedback and the meticulous review and constructive comments on our manuscript. The comments have been invaluable in improving the quality of our manuscript.

In response to the reviewer comments, we have diligently implemented the necessary corrections throughout the manuscript. It is important to note that these revisions may have resulted in changes to line and page numbers. Our commitment to enhancing the clarity and precision of our submission has been paramount in making these adjustments.

Please find below our responses to the reviewers’ comments; the comments are italicized while the responses are highlighted in read. Moreover, the ensuing corrections or changes in the manuscript are tracked for easy recognition.

**Reviewer #1:**

**Comment**

*I appreciate the authors highlighting health disparities by emphasizing the importance of weighing newborn babies, and how it will help health organizations and policy administrations address neonatal complications and mortalities.*

**Response**
No changes required. Many thanks for the compliment.

**Comment**

**Methods**:
*The study conducted both unadjusted and adjusted analyses. In the adjusted analysis, did the authors explore whether there were any interaction effects?*

**Response**
Many thanks for this insightful suggestion. Accordingly, we have included interactions in the adjusted analysis. We interacted place of residence and birthplace, using ANC and birthplace and education and place of residence (see the revised estimates in Table 3 for the results on pages 9 and 10).

**Comments**

*Additionally, did the authors identify any high correlations between independent variables? For example, are women who are wealthy more likely to live in urban areas and also be well-educated?*

**Response**

Thank you for drawing our attention to the need to provide evidence on the extent of collinearity between the independent variables. We performed a diagnostic test for collinearity between the independent variables by looking at the variance inflation factor (VIF) and tolerance indices (TI) of the independent variables (see page 5 lines 20 to 26).

**Comment**

**Results and Discussion:**
• *Page 5, line 21 to 27: I see which African country has the highest and lowest percentage of children weighted at birth. However, I don’t see authors mentioning why that is the case.
• This also raises the question of why the authors did not include the country as an independent variable in the adjusted analysis. Despite the similarities within sub-Saharan countries, health policy and care delivery might differ among these countries, potentially affecting the final response variable, i.e., recording infant weight after birth.*

**Response**

We have addressed this concern by including the country as an independent variable in the adjusted analysis. See table 3 for the results on pages 9 and 10 and page 12 line 20 to 26 for possible explanation for the differences in the percentages of children weighed at birth across the sampled countries. For instance, in 2006, Rwanda launched a national Facility-Based Childbirth Policy (FBCP), which provides free antenatal care and facility-based delivery services. The policy led to an increase in intuitional delivery and other maternal healthcare services. Perhaps, the reason why children in Rwanda are more likely to be weighed compared to children from other countries.

**Comment**
*Table 1 – consider removing the column ‘% of children not weighted at birth’ as it seems redundan*t.

**Response**

Many thanks. This has been duly deleted.

**Comment**
*The numbers in Table 2 don’t add up to 100%. There is a small margin of error for some categories. For example, when adding the percentages for 'weighed' and 'not weighed' for both male and female genders, it does not exactly equal the pooled sample total. The same issue arises for birth type. I encourage the authors to double-check the numbers."*

**Response**

Thanks for drawing our attention to this anomaly. We have made the requisite changes.

We hope our revision will meet your expectations.

Yours faithfully

Edward Nketiah-Amponsah (On behalf of co-authors)
