# Supplementary material for: Factors associated with weighing a child at birth: Evidence from 16 sub-Saharan African countries
Source: PLoS One. 2025 Jul 22;20(7):e0328463. doi: 10.1371/journal.pone.0328463 (PMC12282927; doi:10.1371/journal.pone.0328463)
Supplement: Attachment — Submitted filename: Response Memo to Reviewers Comments_Birthweight Manuscript.docx [file pone.0328463.s008.docx]

**DEPARTMENT OF ECONOMICS**

UNIVERSITY OF GHANA

Telephone: 501485, 500381

*Telegraph & CABLES – UNIVERSITY, LEGON*

*Telex: 2556 UGL.GH*

*Fax: 233-021-501486*

*Email: economics@ug.edu.gh*

**P. O. BOX LG57**

**LEGON, ACCRA**

**GHANA**

February 19, 2025

Dear Editor,

**SUBMISSION OF REVISED MANUSCRIPT FOR PUBLICATION IN PLOS ONE**

We wish to thank you and the reviewers sincerely for your constructive comments on our manuscript titled ‘**Factors associated with weighing a child at birth: evidence from 16 sub-Saharan African countries’** submitted to PLOS ONE for publication consideration. The comments have been invaluable in improving the quality of our manuscript.

In response to the reviewer comments, we have diligently implemented the necessary corrections throughout the manuscript. We have addressed the comments point-by-point as far as feasible. Please find below our responses to the reviewers’ comments; the comments are boldened while the responses are italicized. Moreover, the ensuing corrections or changes in the manuscript are identified by page numbers for easy recognition whenever applicable.

1. **The authors should explain why the year of data collection was not included in the analysis.**

*Response: The model initially included the year variable to account for potential temporal effects. However, it (year) was perfectly collinear with the country variable. Given this collinearity, the year variable was omitted from the final model. We can still show the results with the year variable if deemed necessary.*

2. **Given the hierarchical nature of the data, the authors should clarify why a hierarchical or multilevel model was not used to analyse the data.**

*Response: We acknowledge the hierarchical nature of the data, where individuals are nested within countries. To address this, we accounted for clustering and country-level heterogeneity in the following ways;*

i. *Clustering at the Country Level: We used clustered standard errors at the country level (vce(cluster Country)) to adjust for within-country correlation. This ensures that our standard errors are robust to potential intra-country dependencies.*
ii. *Country-Fixed Effects: We included country-fixed effects (i.Country) in our regression model to control for unobserved country-level characteristics that might influence the outcome variable. This approach allows us to isolate within-country variation while accounting for systematic differences across countries.*
3. **The authors should clarify why health indicators from previous births were not considered.**
   *Response: Health indicators from previous births are only available for women who have had more than one birth. Including these variables would exclude women with only one birth from the analysis. Since our study is not limited to women with multiple births, we chose not to incorporate these indicators to maintain a broader and more representative and consistent sample.*
4. **For each variable in Table 2, the authors should present the percentages of missing data.**

*Response: This has been duly provided in the revised version of the manuscript. See Table 2 (pages 7 and 8)*

5. **The authors should clarify why they did not consider the weight of the data in their analysis.**

*Response: Our analysis accounted for survey sampling weights by specifying the probability weights (pweight=v005) in our regression model. See page 5, line 24.*

6. **The authors should clarify why significant factors like employment status and birth order were excluded from the analysis.**

*Response: We have included employment status (currently working variable) in the revised version of the manuscript. The model did not include birth order due to collinearity with the parity variable. We are happy to include it regardless, if deemed necessary.*

7. **The authors should clarify how they handled missing data.**

*Response: We handled missing data using listwise deletion. Thus, observations with missing values in any of the variables included in the regression model were excluded from the analysis. This approach ensures consistency across all model estimates. See page 5, lines 24 to 27.*

We hope our revisions will meet your kind expectations.

Yours faithfully,

**Prof. Edward Nketiah-Amponsah, PhD** (on behalf of all co-authors)
